# SEMplMe: a tool for integrating DNA methylation effects in transcription factor binding affinity predictions

**DOI:** 10.1186/s12859-022-04865-x

**Published:** 2022-08-04

**Authors:** Sierra S. Nishizaki, Alan P. Boyle

**Affiliations:** 1 Department of Human Genetics, University of Michigan, Ann Arbor, MI 48109 USA; 2 Department of Computational Medicine and Bioinformatics, University of Michigan, Ann Arbor, MI 48109 USA

**Keywords:** DNA methylation, Transcription factor, TFBS, Gene regulation, Noncoding variation, Software, Open-source

## Abstract

**Motivation:**

Aberrant DNA methylation in transcription factor binding sites has been shown to lead to anomalous gene regulation that is strongly associated with human disease. However, the majority of methylation-sensitive positions within transcription factor binding sites remain unknown. Here we introduce SEMplMe, a computational tool to generate predictions of the effect of methylation on transcription factor binding strength in every position within a transcription factor’s motif.

**Results:**

SEMplMe uses ChIP-seq and whole genome bisulfite sequencing to predict effects of methylation within binding sites. SEMplMe validates known methylation sensitive and insensitive positions within a binding motif, identifies cell type specific transcription factor binding driven by methylation, and outperforms SELEX-based predictions for CTCF. These predictions can be used to identify aberrant sites of DNA methylation contributing to human disease.

**Availability and Implementation:**

SEMplMe is available from https://github.com/Boyle-Lab/SEMplMe.

**Supplementary Information:**

The online version contains supplementary material available at 10.1186/s12859-022-04865-x.

## Introduction

DNA methylation is an epigenetic mark as it contributes to changes in the information content of DNA without changing the underlying sequence. The majority of DNA methylation in the human genome occurs at cytosine-phosphate-guanine (CpG) nucleotides. These have long been considered a repressive mark based on early studies of promoters where methylation correlated with transcriptional repression [1]. Methylation at transcription factor binding sites has previously been thought to correlate with the repression of transcription by either disrupting the binding of methylation-sensitive transcription factors or by having no effect on methylation-insensitive transcription factor binding [2]. However, recent high throughput studies have found that methylation within transcription factor binding sites can lead to increased or decreased transcription factor binding dependent on the position within the motif [3, 4]. Recent work has shown that the strength of the effect of methylation on transcription factor binding affinity varies between nucleotides within a single transcription factor motif [3, 4]. It is vital to determine the specific functional impact of methylation within transcription factor binding sites as aberrant methylation is a hallmark of many human diseases, including cancer, schizophrenia, and autism spectrum disorders [5–7]. Methods that can better predict these effects on gene transcription can assist in identifying and prioritizing potentially harmful variations.

The effect of methylation on the binding of individual proteins has been studied in vitro using protein binding microarrays (PBMs) and newer systematic enrichment of ligands by exponential enrichment (SELEX) based methods [8–11]. Both PBMs and SELEX rely on proteins binding to DNA fragments in vitro and may not recapitulate endogenous binding patterns within the genome. A recent study of methylation sensitivity in 542 human transcription factors using a high throughput SELEX method, methyl-SELEX, found 23% of transcription factors were sensitive to methylation, 34% were enhanced by methylation, and 40% were insensitive to methylation [3]. Computational methods to analyze methyl-SELEX data, such as Methyl-Spec-Seq, provide quantitative information on the magnitude and direction of the predicted effect of methylation on transcription factor binding [12]. Additional SELEX-based studies have also observed differences in methylation sensitivity between different positions in a single motif, and is supported by evidence that some bases within transcription factor binding motifs are more correlated with disease compared to others [13, 14]. However, predictions produced from these methods are limited as they rely on in vitro SELEX data and may not reflect binding patterns in a native context. Methods to determine the methylation sensitivity of transcription factors in vivo exist, however they are experimentally rigorous, or do not directly estimate methylation consequence on transcription factor binding, and are therefore challenging to use for broad interpretation [4, 15, 16]. A robust method to study the native context of DNA methylation within transcription factor binding sites using in vivo data is still needed to more accurately model the role these epigenetic marks play on transcriptional regulation.

To address this need, we have adapted SEMpl, a computational genome-wide transcription factor binding affinity prediction method, to incorporate whole genome bisulfite-seq (WGBS) data. This allows our predictions to include the effects of DNA methylation on binding affinity [17]. SEMpl uses open-source in vivo data to generate predictions using transcription factor binding data from ChIP-seq and open chromatin data from DNase-seq for a transcription factor of interest. The results are displayed as a SNP effect matrix providing predictions for every potential base change in a transcription factor’s motif. Our SNP Effect Matrix pipeline with Methylation (SEMplMe) method expands these results by incorporating methylation data from WGBS, generating predictions that encompass the magnitude and direction of change to transcription factor binding for all 4 nucleotide base pairs, and adds two additional nucleotide letters: methylated C (M), and G opposite to a methylated C (W). This new tool provides improved specificity to determine which variants lead to disruption of transcription factor binding by integrating endogenous functional information on methylation states and transcription factor binding, advancing our ability to interrogate and prioritize mutations likely to be associated with human disease.

## Methods

### Usage/accessibility

SEMplMe is open source and can be downloaded from https://github.com/Boyle-Lab/SEMplMe. Precomputed SEMplMe plots are available for more than 70 transcription factors (Additional file 2: Table S1).

### SNP effect matrix pipeline with methylation

**Fig. 1:**
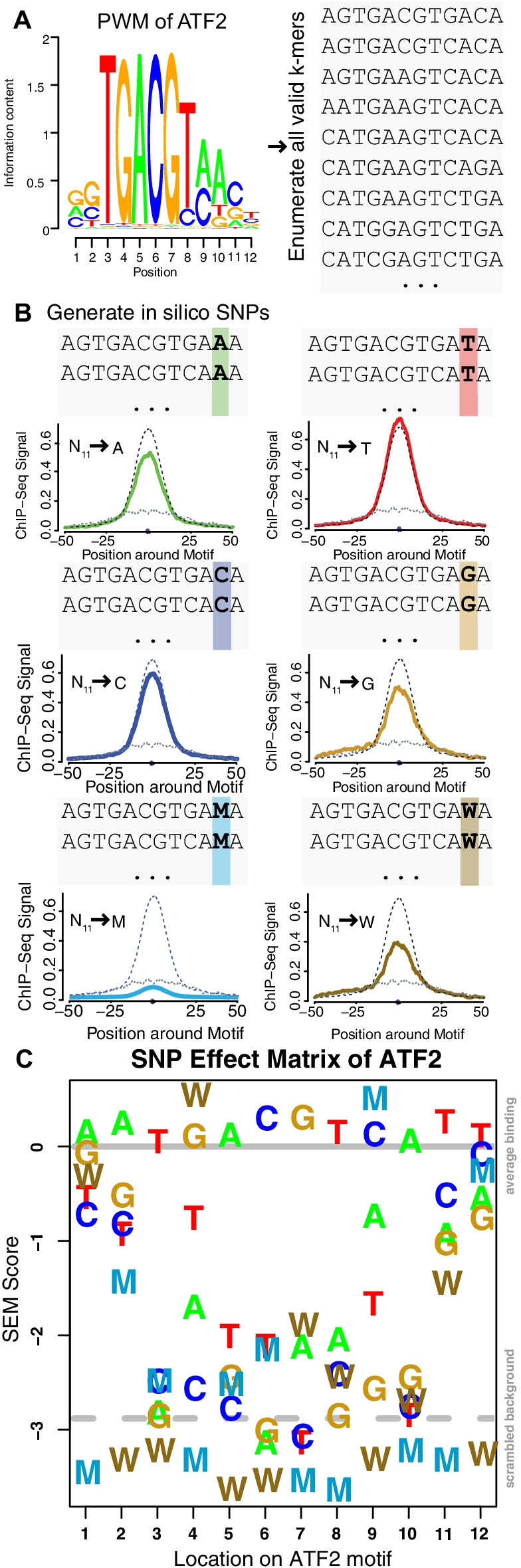
SEM pipeline with methylation predicts the effect of methylation on transcription factor binding affinity. **A** Using SEMpl all kmers with a PWM score under the TFM-*P* VALUE threshold are generated for the given transcription factor [18]. **B** SEMpl then generates all possible ‘in silico variants’ for each position of a transcription factor’s motif. These enumerated kmers are aligned to the genome in regions of open chromatin by DNase-seq, and the average ChIP-seq signal is determined for each alignment to generate SEMpl predictions for each base individually. SEMplMe then expands on this SEMpl output by adding WGBS to divide ChIP-seq signal peaks of C and G into the proportion of their signal affected by DNA methylation using a weighted sum. **C** SEMplMe output is displayed as all 6 nucleotides, including methylated C (M), and G opposite to methylated C (W), at every position along the motif. All values are displayed as log 2 and normalized to an endogenous binding baseline set to 0 (dark gray line). A scrambled baseline is also included (dashed gray line)

SEMplMe functions as an extension of our previously published expectation–maximization method, SEMpl [17]. SEMpl uses an estimation-maximization-like algorithm to predict the consequence variation to binding in transcription factor binding sites using three data types: chromatin immunoprecipitation followed by deep sequencing (ChIP-seq) data, endogenous measures of transcription factor binding genome-wide, DNase I hypersensitive site (DNase-seq) data, a measure of open chromatin genome-wide where transcription factors are known to function; and position weight matrices (PWMs), representing previous knowledge of the binding motif for a transcription factor. SEMpl uses the kmers generated from a given PWM to generate SNP kmer lists by simulating all possible in silico variants along each kmer. These kmers are then aligned to the genome in regions of open chromatin so that their analogous ChIP-seq scores can be averaged for each kmer locus with a shared nucleotide and motif position (i.e. a C in position 3 of the motif). SEMpl used these averaged values to estimate the consequence of all possible variants in a binding site and outputs a matrix of predictions for each of the four nucleotides at each position of a transcription factor’s motif. By including whole genome bisulfite sequencing (WGBS) data to the final output of SEMpl, we have expanded the interpretation of our algorithm to include the contribution of DNA methylation on transcription factor binding (Fig. 1). Starting PWM and cell type used to generate this SEM and all other data shown in figures in this paper can be found in Additional file 2: Table S2.

In order to evaluate the consequence of DNA methylation in transcription factor binding sites we first gathered WGBS for each kmer aligned to the genome containing an in silico SNP. All data shown was generated using matched cell types for ChIP-seq, DNase-seq, and WGBS data. As the vast majority of sites in WGBS data methylation are not binary, the contribution of the proportion of methylation on binding for C and G SNPs at each position within a motif is calculated. Methylation is calculated for each aligned SNP list using the equation: $\mathop \sum \nolimits_{k = 1}^{n} \left( {M_{k} *S_{k} } \right)/n$, where M represents the proportion of methylation for an aligned kmer, S represents the ChIP-seq signal for it’s alignment, and n represents the total number of kmers in the list. Therefore, the equation: $\mathop \sum \nolimits_{k = 1}^{n} \left( {1 - \left( {M_{k} *S_{k} } \right)} \right)/n$ represents the signal contribution of the non-methylated kmer. Using this method, cytosines are divided into methylated and non-methylated components for each position within the motif of a transcription factor. Following this, all 6 nucleotides are included in a SNP effect matrix at each position along the motif of the transcription factor and plotted for an easy to visualize model of transcription factor binding (Fig. 1C).

SEMplMe is written in C++ , perl and R. In addition to a the matrix file (.me.sem) and the pdf of the visualized sem (*_semplot.me.pdf), the output also includes a matrix of standard error (.sterr) and a matrix of total ChIP-seq signal (.me.totals). New alignment and baseline files are also generated for SEMplMe (.me). A quality control file was used, which provides the -log10(P-value) of the average of 100 *t*-tests from 1000 randomly chosen kmers from the signal files versus the scrambled signal files from SEMpl (Additional file 2: Table S1). A threshold of 3.15 was set to report confidence in a SEM plot, with runs falling under this threshold highlighted in red.

### SEMplMe sequence scoring

Scoring a full sequence with SEMplMe can be done in the same manner as PWMs or SEMpl, where the log2 score analogous to the nucleotide of interest at each position is added to reflect the predicted binding score of the sequence. This allows predictions to be made for motifs carrying more than one variant.

### EMSA

Kd values for CEBPB and ATF4 were calculated from a previously published EMSA reaction by densiometric scanning by ImageJ and the Excel Solver Package [9, 19, 20]. All EMSA scores are represented as a ratio to the unmethylated control.

### Correlation with ChIP-seq and illumina 450 k data

All kmers likely to bind CTCF were recovered from the final iteration of SEMpl. For each kmer with at least 50 occurrences, the average ChIP-seq signal and standard error were calculated. Correlation cutoffs for SEMplMe were defined as the scrambled baseline for the final iteration of SEMpl.

Illumina 450 k microarray M-values are previously published and publically available [21]. Microarray probes were mapped to the genome and transcription factor binding to each probe loci was determined using the JASPER CORE 2022 predicted transcription factor binding site database [22]. 61 bp sequences encompassing the predicted CTCF binding sites were scored using CTCF specific matrices from SEMplMe, SEMpl, and Methyl-Spec-seq.

Correlation comparisons were completed using the cocor R package using one tailed-tests and a 0.95 CI [23].

## Results

### SEMplMe provides quantitative predictions based on in vivo measures of binding affinity

SEMplMe integrates endogenous functional data encompassing transcription factor binding, open chromatin, and DNA methylation to provide a quantitative prediction of the effect of methylation on transcription factor binding affinity at every position within a binding motif. By including measures of DNA methylation, SEMplMe is able to calculate the relative average transcription factor binding affinity of methylated genomic sequence by using a weighted sum of ChIP-seq signal and the proportion of methylation at the site from WGBS (Fig. 1). Averaging this signal genome-wide for methylated and unmethylated sequence separately allows for the generation of a quantitative prediction matrix of the effect methylation has on transcription factor binding affinity (Fig. 1C). SEMplMe represents an advancement over currently existing methods as its predictions are generated from in vivo functional data, it is generally accessible without additional experimental work, and the resulting matrix is both quantitative for a single position and across an entire motif.

### SEMplMe recapitulates differences in methylation sensitivity between transcription factors

**Fig. 2:**
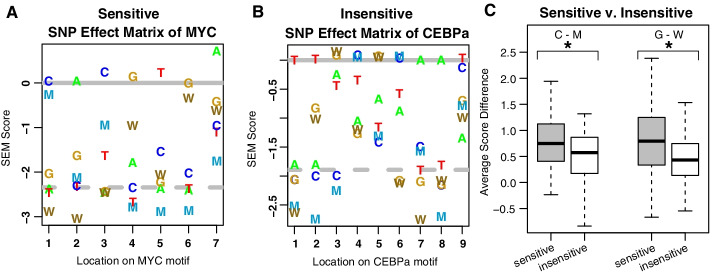
SEMplMe confirms differences in methylated SEM scores for previously classified sensitive versus insensitive transcription factors. **A**. Known methylation sensitive transcription factor MYC shows a large difference between methylated and unmethylated nucleotides at most positions. **B**. Known methylation insensitive transcription factor CEBPa shows very little difference between methylated and unmethylated nucleotides at most positions. For some positions (i.e. position 5), a small increase in binding is predicted for a methylated cytosine. **C**. Transcription factors previously annotated as methylation sensitive and insensitive show a significant difference in methylated (M/W) and non-methylated (C/G) SEM scores (*T*-test C-M *p* –value = 0.007 and G-W *p*-value = 1.32*10^–7^). Error bars represent standard deviation

Transcription factor differences in methylation sensitivity were examined by calculating the absolute difference between methylated and unmethylated bases at each position within SEMplMe matrices for previously classified methylation sensitive and insensitive transcription factors. Methylation sensitive transcription factors examined here include CREB, cMYC, USF, NFkB, E2F, MYC, and ZFX [2, 11, 24, 25]. Methylation insensitive transcription factors examined here include SP1, REST, CEBPa, FOXA1, RXRA, and ARNT2 [2, 8, 11, 25–27]. As expected, transcription factors previously associated with methylation sensitivity show a larger average difference in SEM scores between C and M, and G and W nucleotides compared to transcription factors previously defined as insensitive (Fig. 2). This pattern is driven by methylation sensitivity across an entire motif, with methylation sensitive transcription factor binding sites containing a larger proportion of sensitive loci on average (sensitive = 62%, insensitive 85%, P-value 0.027). Altogether, this suggests that prior definitions of methylation sensitivity and insensitivity may reflect general trends of transcription factor methylation sensitivity.

### DNA methylation drives cell type specific transcription factor binding

**Fig. 3:**
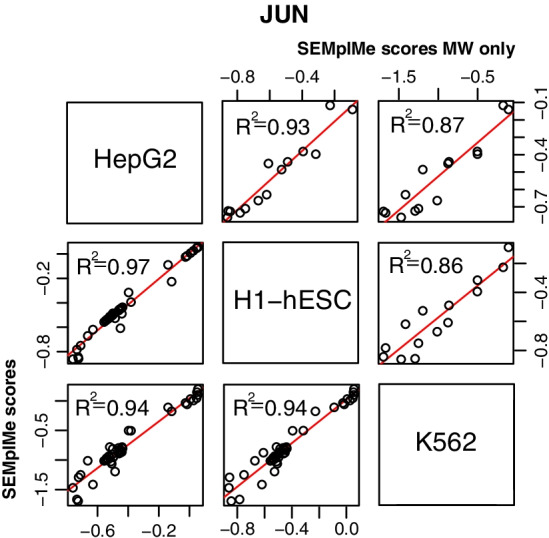
SEMplMe output for JUN varies between cell types. SEM plots vary more between cell types when only considering methylated sites (top right) than methylated and unmethylated sites (bottom left) (correlation comparison: HepG2 v. H1-hESC *p*-value <  = 0.01, HepG2 v. K562 p-value <  = 0.01, H1-hESC v. K562 *p*-value <  = 0.07). This suggests methylation plays a key role in the cell type specificity of the transcription factor JUN

DNA methylation is hypothesized to contribute to cell type specific transcription factor binding by altering the availability of DNA sequence. In support of this, the ChIP-seq and WGBS cell type used in SEMplMe analysis was found to influence the output plot for a transcription factor known to have cell-specific activity. JUN, a transcription factor known to be differentially regulated in HepG2 cells, shows high correlation of SEMplMe outputs for methylated sites (MW) between H1-hESC and K562 cell lines (R^2^ = 0.91), and a reduced correlation to HepG2 (R^2^ = 0.43) (correlation comparison: HepG2 v. H1-hESC *p*-value <  = 0.01, HepG2 v. K562 p-value <  = 0.01, H1-hESC v. K562 *p*-value <  = 0.07) (Fig. 3) [28]. This is supported by MethMotif data, in which JUN shows many more methylated binding sites, most of which fall into a mid- to highly-methylated state in HepG2, as opposed to comparatively few overlapping methylated sites in K562 and H1-hESC [15]. This pattern of reduced correlation was not observed when looking across the entire SEMplMe output, suggesting methylated sites are driving this difference (Additional file 1: Figure S1). Of note, this pattern is not seen for another transcription factor, CEBPB, where the SEMplMe output for methylated sites is highly correlated between all cell types examined (K562, IMR-90, HepG2, and GM12878), suggesting that not all transcription factors are subject to cell type specificity due to methylation differences (Additional file 1: Figure S2 Interestingly, SEMpl data without methylation appears to be primarily cell type agnostic, providing evidence that methylation plays a meaningful role in cell type specificity for only some transcription factors [17].

### SEMplMe validation using in vitro measures of transcription factor binding affinity

**Fig. 4:**
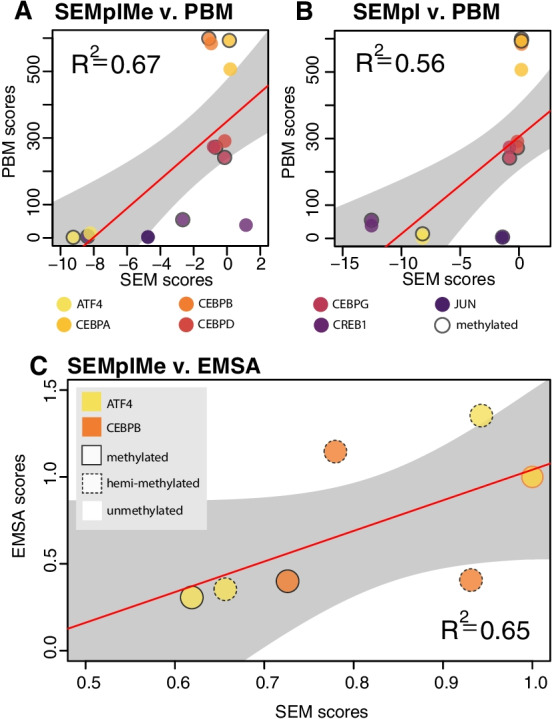
SEMplMe predictions agree with in vitro experimental methods. **A**. SEMplMe agrees with previously published protein binding microarray (PBM) data of methylated and unmethylated binding sites for 8 transcription factors (R^2^ = 0.67) [10]. **B**. SEMpl shows a reduced correlation with PBM data compared to SEMplMe (R^2^ = 0.56), suggesting the addition of methylation data improves methylated sequence predictions.** C**. SEMplMe predictions correlate with previously published electrophoretic mobility shift assay (EMSA) data for methylated, hemi-methylated, and unmethylated binding sites for ATF4 and CEBPB (R^2^ = 0.65) [9]. Grey ribbons represent Pearson’s 0.95 CI

To evaluate SEMplMe on a metric external to ChIP-seq data, our predictions were compared to previously published PBM data, which has been used by previous studies to examine the occupancy of individual transcription factors to potential target sequence in vitro [8, 9]. SEMplMe predictions were compared to microarray Z-scores data from PBMs, which represent transcription factor binding affinity to an individual oligo which is either methylated or unmethylated. The best-bound 8-mers for each of the 8 transcription factors with both PBM Z-scores and SEMplMe scores were assessed. A modest level of agreement was observed between SEMplMe predictions and PBM data for 16 DNA sequences across 8 transcription factors (Fig. 4A) (R^2^ = 0.67) (CEBPA, CEBPB, CEBPD, CREB1, ATF4, JUN, JUND, CEBPG) [9]. This agreement is reduced when using SEMpl scores without methylation (R^2^ = 0.56), suggesting that the inclusion of methylation in our model improves scores for methylated sequences (Fig. 4B) (correlation comparison: *p*-value = 0.33). Discrepancies between SEM predictions and PBM data may be attributed to differences in in vivo versus in vitro methods of generation.

To further functionally validate SEMplMe, data from in vitro electrophoretic mobility shift assays (EMSAs) were utilized to examine our predictions. EMSA data can be used to analyze the binding of transcription factors in the presence of variation in a quantitative manner by comparing dissociation constants between oligos of interest [Zuo, 2017]. Previously published EMSA data was evaluated for two transcription factors, ATF4(CREB) and CEBPB. This measure of in vitro binding showed marginal agreement with our predictions (R^2^ = 0.65) (Fig. 4C) [9]. This observed low agreement is driven entirely by CEBPB which has relatively low correlation with our predictions (R^2^ = 0.17), as opposed to ATF4 (R^2^ = 0.92). CEBPB has been reported to preferentially bind to methylated sequence, thus the discrepancy in predictions has previously been thought to be a result of limited genome methylated sequence availability, a necessity for calculating more accurate predictions in SEMplMe [9]. SEMplMe identified comparatively few methylated sites throughout the genome, leading to a much higher standard deviation for the effect of methylated sites (Additional file 1: Figure S3). This unavailability of methylated sites is consistent with previous data showing methylated CEBPB motifs to bind well in vitro, but poorly in vivo [29].

### SEMplMe predictions are consistent with previous findings for CTCF

**Fig. 5:**
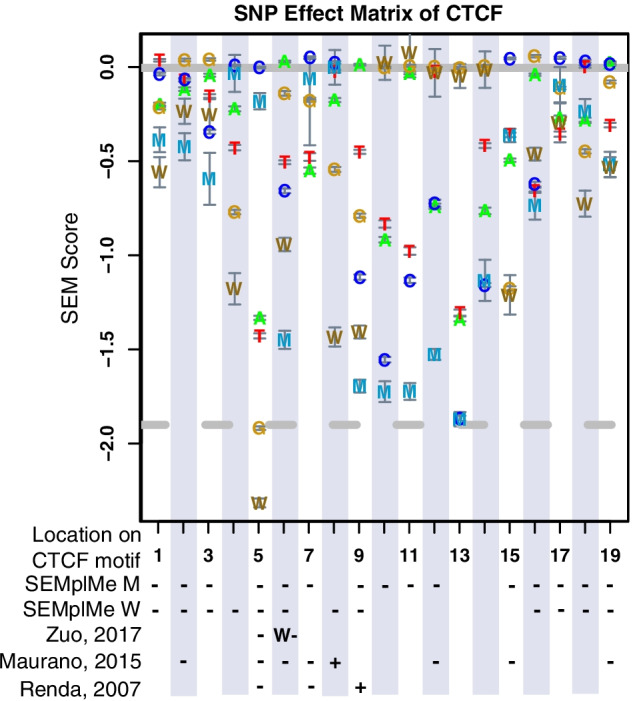
SEMplMe predictions agree with previously published predictions and experimental measures of CTCF binding to methylated sequence. Signs ( ±) found below the SEM plot represent the reduction or increase in binding affinity reported by previous studies at the analogous position. All signs shown without an M or W represent a methylated cytosine (M). Error bars represent standard deviation [4, 12, 32]

CTCF is a well studied transcription factor previously shown to be methylation sensitive [30, 31]. CTCF binding predictions using SEMplMe found the majority of positions to be methylation sensitive for both M and W. Notably, a handful of sites had methylated sequence scores at or slightly above their unmethylated counterparts, and likely represent methylation insensitive positions. These results are consistent with CTCF’s role as a methylation sensitive transcription factor. As CTCF is widely used in research studies, its binding to sites containing methylated positions within its motif have been previously surveyed by a variety of methodologies, including qualitative EMSA, observation of binding following demethylation of cells, and SELEX-based methods [4, 12, 30, 32]. When SEMplMe results were compared to measures of binding at individual positions within the CTCF motif, a general agreement was observed for the direction of binding for all positions predicted to decrease binding affinity (Fig. 5). Though the majority of sites identified by previous studies within the CTCF motif were found to be overwhelmingly methylation sensitive, two sites were predicted to lead to increased binding affinity when methylated. Though SEMplMe did not identify these positions, one site overlaps a SEMplMe position consistent with methylation insensitivity, and the other was found to not significantly increase binding by all prior studies [4]. Overall, our predictions are consistent with previous studies of CTCF binding direction.

**Fig. 6:**
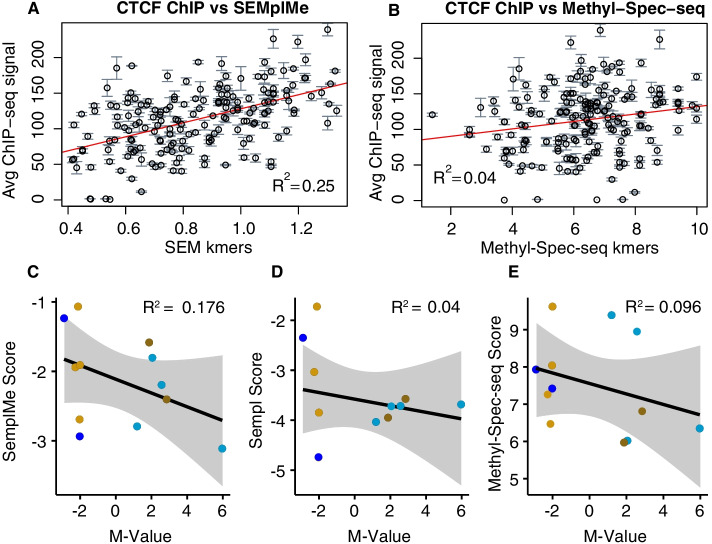
SEMplMe has higher correlation with in vivo CTCF binding than Methyl-Spec-seq. A. **A** and **B**. SEMplMe outperforms Methyl-Spec-seq when comparing to CTCF ChIP-seq scores for whole kmers, including methylated sites (SEMplMe R^2^ = 0.25, Methyl-Spec-seq R^2^ = 0.04, correlation comparison *p*-value <  = 0.01). **C**–**E**. SEMplMe is more closely inversely correlated with methylation occupancy in CTCF binding sites from an Illumina 450 k microarray dataset compared to SEMpl without methylation, and Methyl-Spec-seq (SEMplMe R^2^ = 0.176, SEMpl R^2^ = 0.04, Methyl-Spec-seq R^2^ = 0.096, correlation comparison *p*-values > 0.132). Grey ribbons represent Pearson’s 0.95 CI

Correlation between the entirety of the CTCF matrices generated by SEMplMe and the recently published Methyl-Spec-seq method, which uses in vitro SELEX to predict methylation effects on transcription factor binding affinity, was assayed (R^2^ = 0.56) (Additional file 1: Figure S4)[12]. SEMplMe outperformed Methyl-Spec-seq by performance comparison when comparing scores across entire kmers to their average ChIP-seq signal (SEMplMe R^2^ = 0.25, Methyl-Spec-seq R^2^ = 0.04, correlation comparison p-value <  = 0.01) (Fig. 6A and B). The kmer set used is associated with active CTCF binding and includes both methylated and unmethylated sequences. Additionally, we compared SEMplMe SEM scores, SEMpl SEM scores without methylation, and Methyl-Spec-seq ePWM scores to in vitro metrics of CTCF protein binding by analyzing all motifs corresponding to CTCF, including to 6 methylated and 6 unmethylated oligos, from a previously published Illumina 450 k microarray. In doing so we found SEMplMe predictions to have a more robust inverse correlation with methylation occupancy (SEMplMe R^2^ = 0.176, SEMpl R^2^ = 0.04, MethylSpec-seq R^2^ = 0.096, correlation comparison *p*-values > 0.132) (Fig. 6C–E) [21]. This is expected, as CTCF is predicted to be methylation sensitive along the majority of its motif. Interestingly, though no statistical differences in correlation comparisons were noted with this small sample size, both tools implementing methylation data outperformed SEMpl without methylation, supporting the importance of including measures of methylation in determining transcription factor binding disruption in the presence of methylation. Altogether, this provides further evidence that predictions of change to transcription factor binding affinity perform better when generated from in vivo data, rather than in vitro data such as from SELEX methods.

## Discussion

SEMplMe is poised to advance our understanding of the effects of methylation on transcription factor binding affinity through its generation of quantitative predictions using in vivo functional data. SEMplMe will both improve our ability to predict putative disease loci affected by aberrant DNA methylation and increase predictions of transcription factor binding affinity in general [25]. This is expected to hold true regardless of whether reduced methylation in a transcription factor’s motif contributes to its binding or is caused by its binding [33]. The nucleotide W was included to capture not just position dependent, but stand dependent methylation, as strand specificity due to hemi-methylation has previously been found to influence transcription factor binding [12]. This is likely driven by changes in DNA structure.

SEMplMe has similar limitations to its predecessor SEMpl, such as a dependence on available ChIP-seq, DNase-seq, and WGBS data. It is further restricted by the limited number of methylated sites in the genome available for use in generating models of binding. In instances where few sequence specific sites also contain methylation, our measure of standard deviation increases considerably. Though the low confidence in these sites can be visualized by error bars, predictions of methylation at these loci are limited. Cell type should be carefully considered before running SEMplMe for optimal predictions as cell type specificity contributes to the final SEMplMe plot, and methylation sensitivity has been previously found to be paralog specific [13]. Additional cell type specific factors such as transcription factor co-binding may also affect the final SEMplMe plot as seen for MYC in SEMpl, though is expected to be rare due to the genome-wide nature of the predictions [17]. Starting PWMs should also be carefully considered for transcription factors known to recognize different methylated and unmethylated motifs [8].

The inclusion of CpG methylation provides additional information to help fully understand context-specific transcription factor binding. However, the addition of more nuanced molecular mechanisms that contribute to transcription factor binding are likely to further improve our predictions. This includes additional types of DNA methylation, such as hydroxymethylation and nonCpG methylation, as well as measures of structural changes to the genome [10, 13, 34–36].

## Conclusions

DNA methylation is a key epigenetic mark known to act in a regulatory capacity, allowing transcription factors to bind in a cell-type specific manner. Counter to the idea that all methylation is able to disrupt transcription factor binding, recent studies have revealed that certain methylated loci impact binding more than others. Predicting the locations of these methylation sensitive loci and quantifying the effect of methylation on transcription factor binding affinity is challenging. Here we introduce an expansion to our previously released software SEMpl, called SEMplMe, which integrates predictions of the effect of cytosine methylation on transcription factor binding affinity based on WGBS data. These predictions agree with in vitro data of transcription factor binding, are cell-type specific, and show a general agreement with data from transcription factors previously annotated as methylation sensitive and insensitive.

The improved predictions provided by SEMplMe will contribute to a better understanding of the key positions within transcription factor binding sites affected by DNA methylation. These predictions are accessible and can be generated without undertaking methylation specific binding assays. This advancement is central to improving our ability to prioritize mutations associated with aberrant methylation contributing to human disease.

## Supplementary Information

**Additional file 1 Fig. S1**. SEMplMe output between cell types versus SEMpl without methylation. SEM plots show more variance between cell types for SEMplMe (top right) than SEMpl without methylation (bottom left). Pairwise correlation comparisons: p-values < 0.001.** Fig. S2**. CEBPB SEM output between cell types. SEM plots show little variance between cell types when considering only methylated sites (top right) or both methylated and unmethylated sites (bottom left) for CEBPB. This suggests methylation does not play a large role in cell type specificity for CEBPB. Pairwise correlation comparisons: p-values < 0.0001.** Fig. S3**. Total number of kmers for each nucleotide in the SEM of CEBPB. A. SEM plot of CEBPB with error bars representing standard deviation. B. Counts of mapped kmers in the genome for each nucleotide at each position. These counts are inversely proportional to the standard deviation seen in the SEM.** Fig. S4**. Correlation of CTCF matrices between SEMplMe and Methyl-Spec-seq show a modest agreement (R2=0.56).

**Additional file 2 Table S1** Data availability for ChIP-set, WGBS, and DNase-seq used in this study along with empirical p-value estimation for each ChIP-seq dataset (see Methods)** Table S2**. Starting PWM and cell type used to generate SEMs and all other data shown in figures in this paper.

## Data Availability

The SEMplMe datasets generated during the current study are available on the project github page https://github.com/Boyle-Lab/SEMplMe

## Abbreviations

CpG: Cytosine-phosphate-guanine
PBMs: Protein binding microarrays
SELEX: Systematic enrichment of ligands by exponential enrichment
WGBS: Whole genome bisulfite-seq
SEMplMe: SNP effect matrix pipeline with methylation
ChIP-seq: Chromatin immunoprecipitation followed by deep sequencing
DNase-seq: DNase I hypersensitive site sequencing
EMSA: Electrophoretic mobility shift assay

## References

[1] Bird AP (1986). CpG-rich islands and the function of DNA methylation. Nature.

[2] Tate PH, Bird AP (1993). Effects of DNA methylation on DNA-binding proteins and gene expression. Curr Opin Genet Dev.

[3] Yin Y, Morgunova E, Jolma A, Kaasinen E, Sahu B, Khund-Sayeed S, Das PK, Kivioja T, Dave K, Zhong F, Nitta KR (2017). Impact of cytosine methylation on DNA binding specificities of human transcription factors. Science.

[4] Maurano MT, Wang H, John S, Shafer A, Canfield T, Lee K, Stamatoyannopoulos JA (2015). Role of DNA Methylation in Modulating Transcription Factor Occupancy. Cell Rep.

[5] Robertson KD (2005). DNA methylation and human disease. Nat Rev Genet.

[6] Gavin DP, Sharma RP (2010). Histone modifications, DNA methylation, and schizophrenia. Neurosci Biobehav Rev.

[7] Jiang Y-H, Sahoo T, Michaelis RC, Bercovich D, Bressler J, Kashork CD, Liu Q, Shaffer LG, Schroer RJ, Stockton DW, Spielman RS, Stevenson RE, Beaudet AL (2004). A mixed epigenetic/genetic model for oligogenic inheritance of autism with a limited role for UBE3A. Am J Med Genet A.

[8] Hu S, Wan J, Su Y, Song Q, Zeng Y, Nguyen HN, Shin J, Cox E, Rho HS, Woodard C, Xia S (2013). DNA methylation presents distinct binding sites for human transcription factors. Elife.

[9] Mann IK, Chatterjee R, Zhao J, He X, Weirauch MT, Hughes TR, Vinson C (2013). CG methylated microarrays identify a novel methylated sequence bound by the CEBPB|ATF4 heterodimer that is active in vivo. Genome Res.

[10] Tillo D, Ray S, Syed K-S, Gaylor MR, He X, Wang J, Assad N, Durell SR, Porollo A, Weirauch MT, Vinson C (2017). The Epstein-Barr Virus B-ZIP protein Zta recognizes specific DNA sequences containing 5-Methylcytosine and 5-hydroxymethylcytosine. Biochemistry.

[11] Zhu H, Wang G, Qian J (2016). Transcription factors as readers and effectors of DNA methylation. Nat Rev Genet.

[12] Zuo Z, Roy B, Chang YK, Granas D, Stormo GD (2017). Measuring quantitative effects of methylation on transcription factor–DNA binding affinity. Sci Adv.

[13] Kribelbauer JF, Laptenko O, Chen S, Martini GD, Freed-Pastor WA, Prives C, Mann RS, Bussemaker HJ (2017). Quantitative analysis of the DNA methylation sensitivity of transcription factor complexes. Cell Rep.

[14] Umer HM, Cavalli M, Dabrowski MJ, Diamanti K, Kruczyk M, Pan G, Komorowski J, Wadelius C (2016). A significant regulatory mutation burden at a high-affinity position of the CTCF motif in gastrointestinal cancers. Hum Mutat.

[15] Xuan Lin QX, Sian S, An O, Thieffry D, Jha S, Benoukraf T (2019). MethMotif: an integrative cell specific database of transcription factor binding motifs coupled with DNA methylation profiles. Nucleic Acids Res.

[16] Wang G, Luo X, Wang J, Wan J, Xia S, Zhu H, Qian J, Wang Y (2018). MeDReaders: a database for transcription factors that bind to methylated DNA. Nucleic Acids Res.

[17] Nishizaki SS, Ng N, Dong S, Porter RS, Morterud C, Williams C, Asman C, Switzenberg JA, Boyle AP (2020). Predicting the effects of SNPs on transcription factor binding affinity. Bioinformatics.

[18] Touzet H, Varré J-S (2007). Efficient and accurate p-value computation for position weight matrices. Algorithms Mol Biol.

[19] Aghera N, Earanna N, Udgaonkar JB (2011). Equilibrium unfolding studies of monellin: the double-chain variant appears to be more stable than the single-chain variant. Biochemistry.

[20] Schneider CA, Rasband WS, Eliceiri KW (2012). NIH image to image J: 25 years of image analysis. Nat Methods.

[21] Reinius LE, Acevedo N, Joerink M, Pershagen G, Dahlén S-E, Greco D, Söderhäll C, Scheynius A, Kere J (2012). Differential DNA methylation in purified human blood cells: implications for cell lineage and studies on disease susceptibility. PLoS ONE.

[22] Castro-Mondragon JA, Riudavets-Puig R, Rauluseviciute I, Lemma RB, Turchi L, Blanc-Mathieu R, Lucas J, Boddie P, Khan A, Manosalva Pérez N, Fornes O, Leung TY, Aguirre A, Hammal F, Schmelter D, Baranasic D, Ballester B, Sandelin A, Lenhard B, Vandepoele K, Wasserman WW, Parcy F, Mathelier A (2022). JASPAR 2022: the 9th release of the open-access database of transcription factor binding profiles. Nucleic Acids Res.

[23] PLOS One Staff (2015). Correction: cocor: a comprehensive solution for the statistical comparison of correlations. PLoS ONE.

[24] Weng Y-L, An R, Shin J, Song H, Ming G-L (2013). DNA modifications and neurological disorders. Neurotherapeutics.

[25] Stadler MB, Murr R, Burger L, Ivanek R, Lienert F, Schöler A, van Nimwegen E, Wirbelauer C, Oakeley EJ, Gaidatzis D, Tiwari VK, Schübeler D (2011). DNA-binding factors shape the mouse methylome at distal regulatory regions. Nature.

[26] Zhang D, Wu B, Wang P, Wang Y, Lu P, Nechiporuk T, Floss T, Greally JM, Zheng D, Zhou B (2017). Non-CpG methylation by DNMT3B facilitates REST binding and gene silencing in developing mouse hearts. Nucleic Acids Res.

[27] Bartke T, Vermeulen M, Xhemalce B, Robson SC, Mann M, Kouzarides T (2010). Nucleosome-interacting proteins regulated by DNA and histone methylation. Cell.

[28] Baichwal VR, Park A, Tjian R (1992). The cell-type-specific activator region of c-Jun juxtaposes constitutive and negatively regulated domains. Genes Dev.

[29] Moll JR, Acharya A, Gal J, Mir AA, Vinson C (2002). Magnesium is required for specific DNA binding of the CREB B-ZIP domain. Nucleic Acids Res.

[30] Bell AC, Felsenfeld G (2000). Methylation of a CTCF-dependent boundary controls imprinted expression of the Igf2 gene. Nature.

[31] Stadnick MP, Pieracci FM, Cranston MJ, Taksel E, Thorvaldsen JL, Bartolomei MS (1999). Role of a 461-bp G-rich repetitive element in H19 transgene imprinting. Dev Genes Evol.

[32] Renda M, Baglivo I, Burgess-Beusse B, Esposito S, Fattorusso R, Felsenfeld G, Pedone PV (2007). Critical DNA binding interactions of the insulator protein CTCF: a small number of zinc fingers mediate strong binding, and a single finger-DNA interaction controls binding at imprinted loci. J Biol Chem.

[33] Han L, Lin IG, Hsieh CL (2001). Protein binding protects sites on stable episomes and in the chromosome from de novo methylation. Mol Cell Biol.

[34] Spruijt CG, Gnerlich F, Smits AH, Pfaffeneder T, Jansen PWTC, Bauer C, Münzel M, Wagner M, Müller M, Khan F, Eberl HC, Mensinga A, Brinkman AB, Lephikov K, Müller U, Walter J, Boelens R, van Ingen H, Leonhardt H, Carell T, Vermeulen M (2013). Dynamic readers for 5-(hydroxy)methylcytosine and its oxidized derivatives. Cell.

[35] Wu X, Zhang Y (2017). TET-mediated active DNA demethylation: mechanism, function and beyond. Nat Rev Genet.

[36] Mathelier A, Xin B, Chiu T-P, Yang L, Rohs R, Wasserman WW (2016). DNA Shape features improve transcription factor binding site predictions in vivo. Cell Syst.

