# Supplementary material for: SEMplMe: a tool for integrating DNA methylation effects in transcription factor binding affinity predictions
Source: BMC Bioinformatics. 2022 Aug 4;23:317. doi: 10.1186/s12859-022-04865-x (PMC9351228; doi:10.1186/s12859-022-04865-x)
Supplement: Supplementary file 1 — Additional file 1 Fig. S1. SEMplMe output between cell types versus SEMpl without methylation. SEM plots show more variance between cell types for SEMplMe (top right) than SEMpl without methylation (bottom left). Pairwise correlation comparisons: p-values < 0.001. Fig. S2. CEBPB SEM output between cell types. SEM plots show little variance between cell types when considering only methylated sites (top right) or both methylated and unmethylated sites (bottom left) for CEBPB. This suggests methylation does not play a large role in cell type specificity for CEBPB. Pairwise correlation comparisons: p-values < 0.0001. Fig. S3. Total number of kmers for each nucleotide in the SEM of CEBPB. A. SEM plot of CEBPB with error bars representing standard deviation. B. Counts of mapped kmers in the genome for each nucleotide at each position. These counts are inversely proportional to the standard deviation seen in the SEM. Fig. S4. Correlation of CTCF matrices between SEMplMe and Methyl-Spec-seq show a modest agreement (R2=0.56). [file 12859_2022_4865_MOESM1_ESM.docx]

**Supplementary Information for**

**SEMplMe: A tool for integrating DNA methylation effects in transcription factor binding affinity predictions**

Sierra S Nishizaki^1,2^, Alan P Boyle^1,2,*^

1. Department of Human Genetics

University of Michigan

Ann Arbor, Michigan USA

1. Department of Computational Medicine and Bioinformatics

University of Michigan

Ann Arbor, Michigan USA

*corresponding author: [apboyle@umich.edu](mailto:apboyle@umich.edu)

**Figure S1.** SEMplMe output between cell types versus SEMpl without methylation. SEM plots show more variance between cell types for SEMplMe (top right) than SEMpl without methylation (bottom left). Pairwise correlation comparisons: p-values < 0.001.

**Figure S2.** CEBPB SEM output between cell types. SEM plots show little variance between cell types when considering only methylated sites (top right) or both methylated and unmethylated sites (bottom left) for CEBPB. This suggests methylation does not play a large role in cell type specificity for CEBPB. Pairwise correlation comparisons: p-values < 0.0001.

**Figure S3.** Total number of kmers for each nucleotide in the SEM of CEBPB. A. SEM plot of CEBPB with error bars representing standard deviation. B. Counts of mapped kmers in the genome for each nucleotide at each position. These counts are inversely proportional to the standard deviation seen in the SEM.

**Figure S4.** Correlation of CTCF matrices between SEMplMe and Methyl-Spec-seq show a modest agreement (R^2^=0.56).

**Table S1.** Data availability for ChIP-set, WGBS, and DNase-seq used in this study along with empirical p-value estimation for each ChIP-seq dataset (see Methods)

**Table S2.** Starting PWM and cell type used to generate SEMs and all other data shown in figures in this paper.


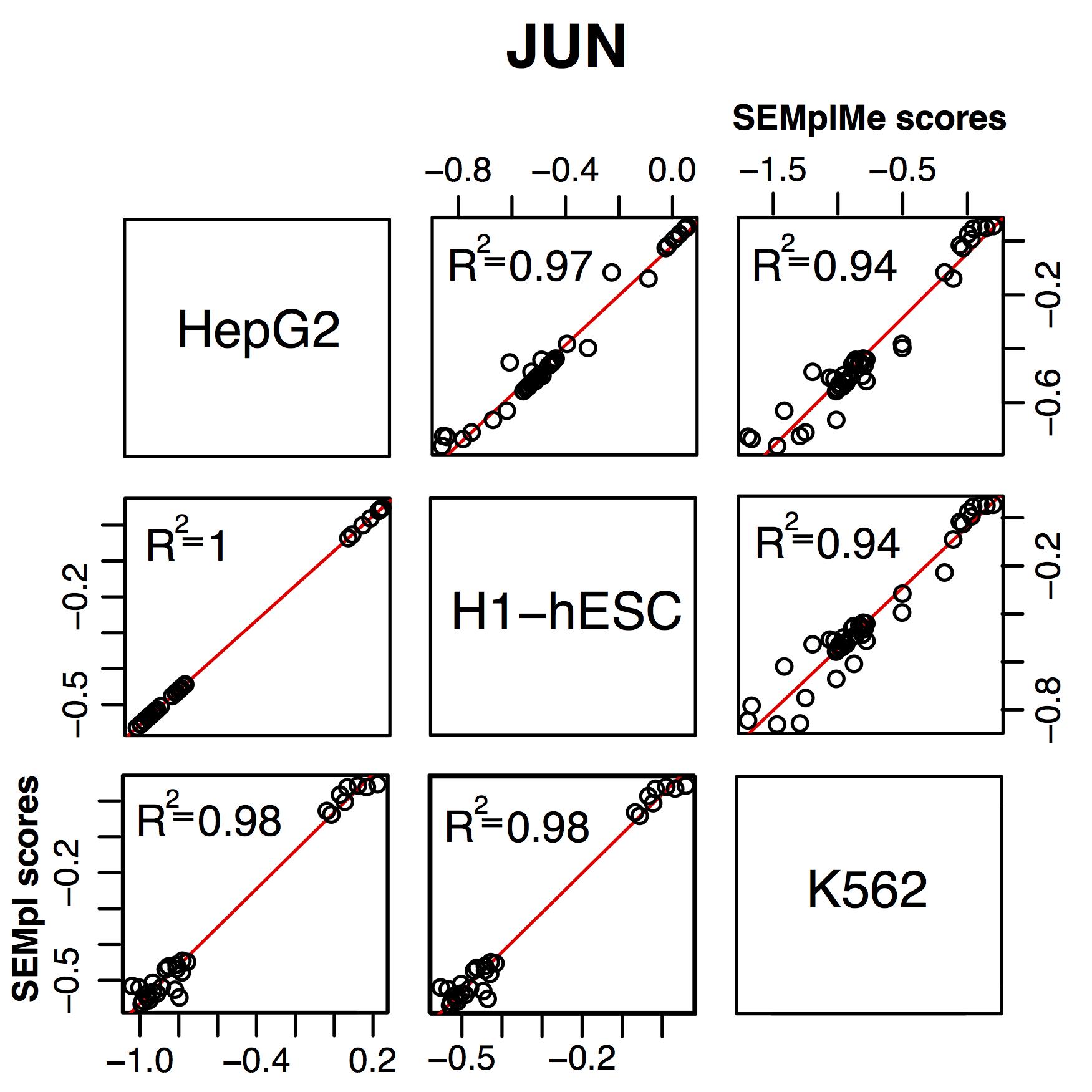


Supplementary Figure 1. SEMplMe output between cell types versus SEMpl without methylation. SEM plots show more variance between cell types for SEMplMe (top right) than SEMpl without methylation (bottom left). Pairwise correlation comparisons: p-values < 0.001.


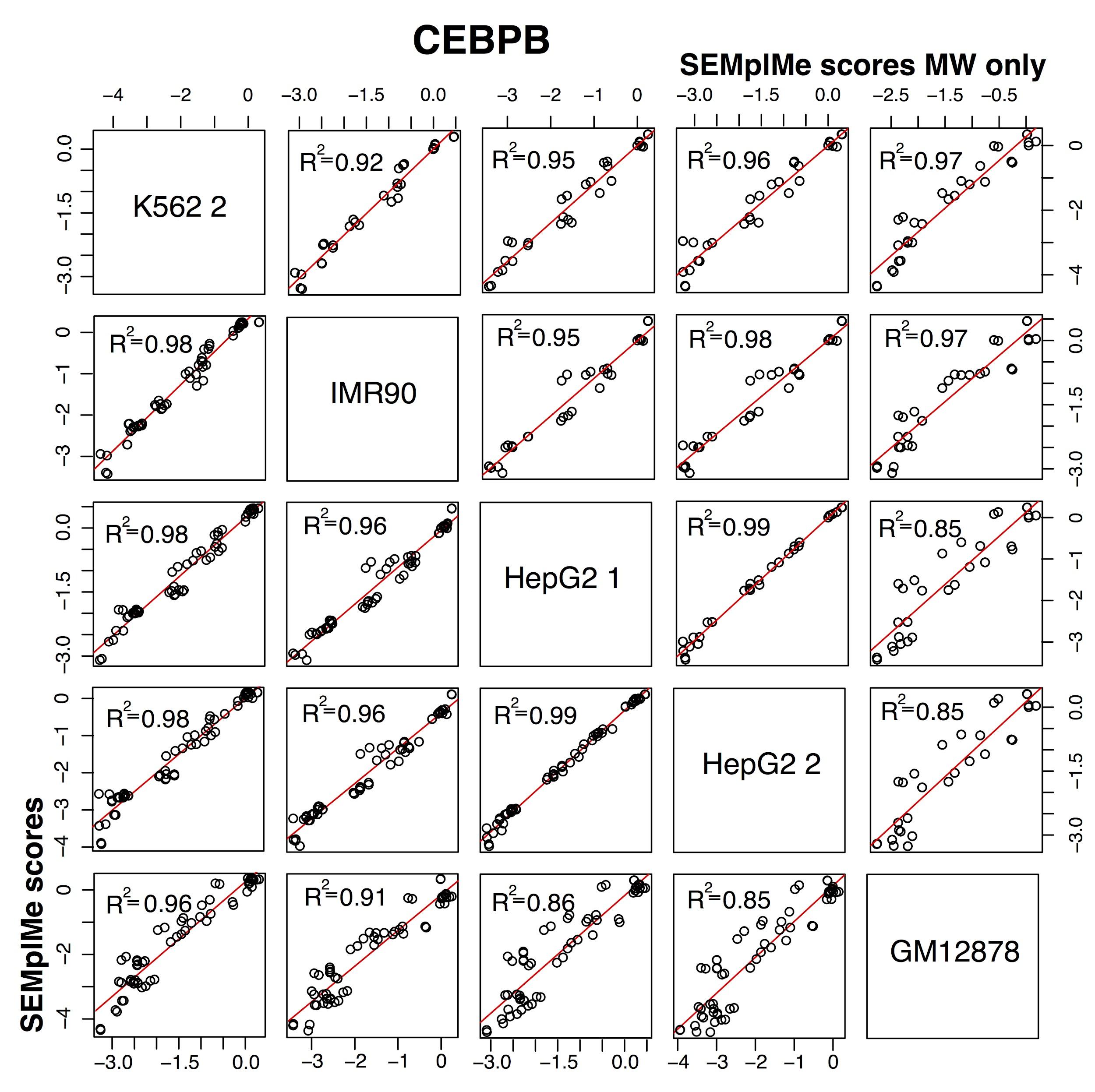


Supplementary Figure 2. CEBPB SEM output between cell types. SEM plots show little variance between cell types when considering only methylated sites (top right) or both methylated and unmethylated sites (bottom left) for CEBPB. This suggests methylation does not play a large role in cell type specificity for CEBPB. Pairwise correlation comparisons: p-values < 0.0001.


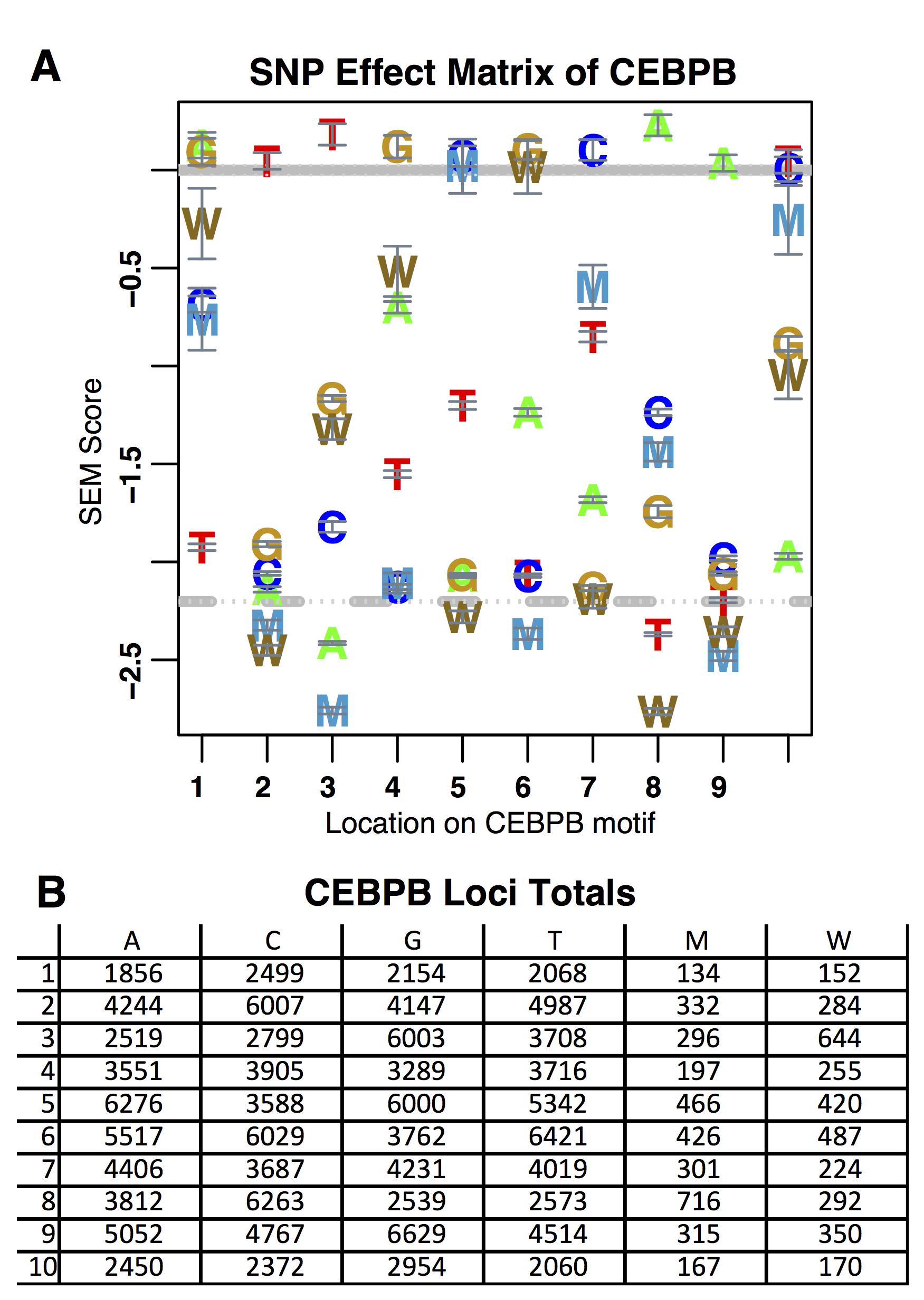


Supplementary Figure 3. Total number of kmers for each nucleotide in the SEM of CEBPB. A. SEM plot of CEBPB with error bars representing standard deviation. B. Counts of mapped kmers in the genome for each nucleotide at each position. These counts are inversely proportional to the standard deviation seen in the SEM.


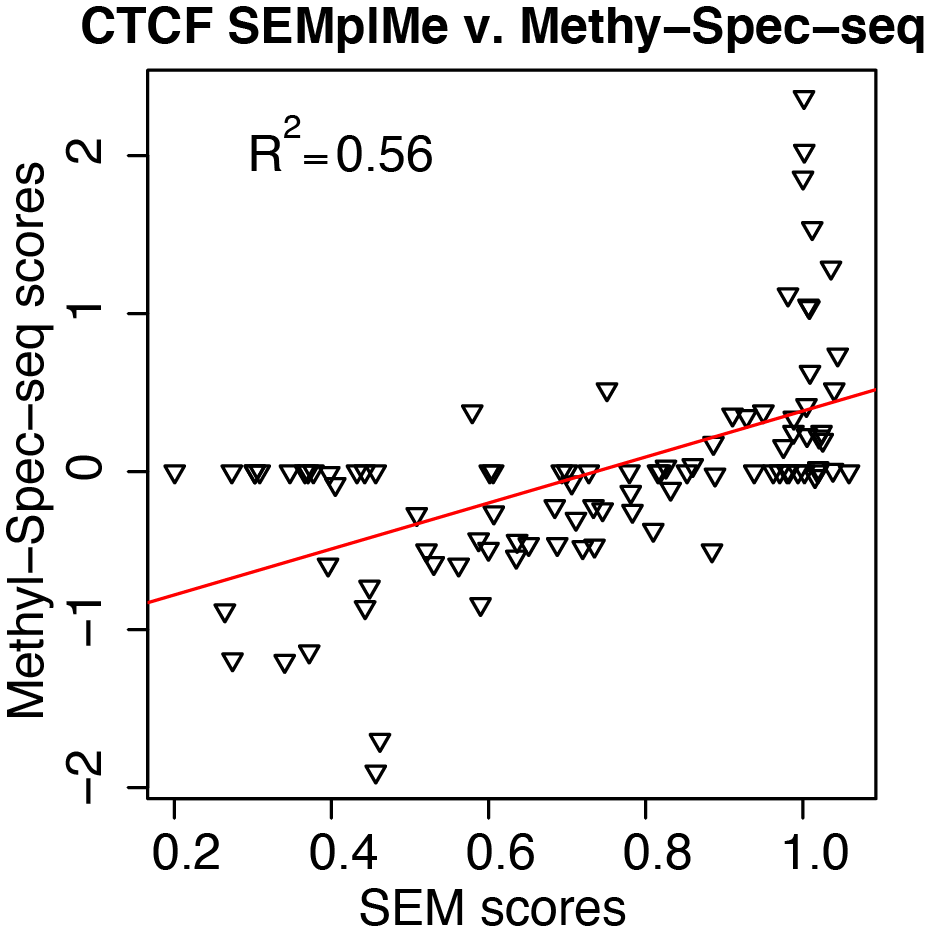


Supplementary Figure 4. Correlation of CTCF matrices between SEMplMe and Methyl-Spec-seq show a modest agreement (R^2^=0.56).
